# Wild Birds and Increased Transmission of Highly Pathogenic Avian Influenza (H5N1) among Poultry, Thailand

**DOI:** 10.3201/eid1706.100880

**Published:** 2011-06

**Authors:** Juthatip Keawcharoen, Jan van den Broek, Annemarie Bouma, Thanawat Tiensin, Albert D.M.E Osterhaus, Hans Heesterbeek

**Affiliations:** Author affiliations: Erasmus Medical Center, Rotterdam, the Netherlands (J. Keawcharoen, A.D.M.E. Osterhaus);; Utrecht University, Utrecht, the Netherlands (J. van den Broek, A. Bouma, T. Tiensin, H. Heesterbeek)

**Keywords:** wild birds, poultry, influenza A virus, subtype H5N1, a nonhomogeneous birth and death statistical model, reproductive power, epidemiology, Thailand, research

## Abstract

Since the outbreaks of highly pathogenic avian influenza (HPAI) subtype H5N1 virus, wild birds have been suspected of transmitting this virus to poultry. On January 23, 2004, the Ministry of Public Health in Thailand informed the World Health Organization of an avian influenza A (H5N1) outbreak. To determine the epidemiology of this viral infection and its relation to poultry outbreaks in Thailand from 2004 through 2007, we investigated how wild birds play a role in transmission. A total of 24,712 serum samples were collected from migratory and resident wild birds. Reverse transcription PCR showed a 0.7% HPAI (H5N1) prevalence. The highest prevalence was observed during January–February 2004 and March–June 2004, predominantly in central Thailand, which harbors most of the country’s poultry flocks. Analysis of the relationship between poultry and wild bird outbreaks was done by using a nonhomogeneous birth and death statistical model. Transmission efficiency among poultry flocks was 1.7× higher in regions with infected wild birds in the given or preceding month. The joint presence of wild birds and poultry is associated with increased spread among poultry flocks.

## Abstract 2

Wild birds are associated with increased virus spread to poultry.

Avian influenza is a viral disease of poultry and is distributed worldwide. The virus is classified based on 2 surface proteins, the hemagglutinin (HA) protein (H1–H16) and the neuraminidase (NA) protein (N1–N9), which can be found in numerous combinations (1). All H and N subtypes can be found as low pathogenic avian influenza virus strains in aquatic wild birds, which are assumed to be the main reservoirs outside poultry (2,3). Occasionally, low pathogenic avian influenza virus strains are introduced into domestic poultry flocks with no clinical signs or only mild clinical consequences, but strains carrying the H5 or H7 gene can mutate into highly pathogenic avian influenza (HPAI) strains that cause high death rates in domestic poultry (4) and, occasionally, in migratory birds (5,6). Because of the devastating effect of HPAI outbreaks in commercial poultry, all outbreaks caused by H5 and H7 subtypes are notifiable (7).

Currently, a HPAI virus strain of subtype H5N1 is circulating in many countries in Eurasia and Africa, causing high death rates in poultry, substantial economic losses, and human deaths. The strain was first identified in Southeast Asia in 1996 and has since spread to 63 countries in Asia, Europe, Africa, and the Middle East (7). Millions of domestic poultry died from the effects of the disease or from culling efforts to control the spread of the virus (1,2,8,9). The spread of the HPAI (H5N1) virus from Southeast Asia to Russia, Europe, and Africa was assumed to originate from a virus source at Qinghai Lake, People’s Republic of China (6,10). Therefore, migratory birds were considered to be responsible for long distance dispersal of the virus (11–13).

In Thailand, 7 waves of HPAI (H5N1) virus outbreaks have occurred since January 2004. Poultry and wild bird populations in 1,417 villages in 60 of the 76 provinces were affected, and >62 million birds died or were culled to prevent further transmission (14–16). Introduction of the virus into poultry flocks is considered to be possible through infected wild birds. Additional insight on the basis of quantitative data into the role of wild birds would be necessary to further develop control measures and surveillance programs.

Relatively little effort has been made to quantify the association between infection in wild birds and outbreaks in poultry flocks, most likely because of the lack of data on infection in wild birds. Recently, a preliminary study was carried out that analyzed the prevalence of HPAI (H5N1) infection in wild birds in Thailand (14). In that study, 6,263 pooled surveillance samples from wild birds in Thailand, collected from January 2004 through December 2007, were tested for evidence of infection. Testing indicated that prevalence patterns in wild birds mirrored outbreaks among poultry; however, the association was not proven or quantified. We studied extensive data on 24,712 wild birds, sampled and analyzed from 2004 through 2007 in Thailand, to quantify the possible effect of infection in wild birds on the spread of the infection among poultry flocks.

## Materials and Methods

### Data Collection

Data about subtype H5N1 infections in wild bird populations were provided by the National Institute of Animal Health of Thailand, Regional Veterinary Research and Development Centers, the Veterinary Science faculty of Mahidol University, and the Department of Livestock Development, Thailand. A total of 24,712 wild bird samples were collected from January 2004 through December 2007. During 2004–2006, sampling was part of a general countrywide surveillance program; in 2007, sampling was targeted specifically at areas where outbreaks in poultry had occurred.

Sampling methods have been described previously (14,16,17). Wild birds were either trapped by using baited traps, hand nets, or mist nets, or shot. Tracheal/oropharyngeal swabs and cloacal swabs of live birds and bird carcasses were collected from active surveillance (sampling of healthy wild birds) and passive surveillance (sampling of sick or dead birds). Swab samples were collected in viral transport media, stored at 4^o^C, and shipped to the laboratory, where they were stored at −80^o^C until further analysis could be done.

### Virus Detection

Methods used for antigen detection have been described by Tiensin et al. (16) and Siengsanan et al. (14). Supernatants from homogenized tissue and swab samples were filtrated and inoculated in 11-day-old embryonated chicken eggs or MDCK cell cultures. After incubation at 37^o^C for 3 days, allantoic fluid was harvested. The inoculated MDCK cell culture was observed daily for cytopathic effect, and supernatant fluid was harvested by day 4, even if no cytopathic effect was observed. Viruses were initially identified in allantoic fluids or culture supernatants by the HA assay according to World Health Organization recommendations (14). Negative samples were inoculated 2 additional times in embryonated chicken eggs before specimens were confirmed as negative.

RNA from positive samples acquired from virus culture was extracted by using a viral RNA extraction kit (QIAGEN, Valencia, California, USA), according to the manufacturer’s instructions. Reverse transcription PCR (RT-PCR) was performed by using a 1-step RT-PCR kit (QIAGEN) to identify the subtype, according to the manufacturer’s instructions. Primers for RT of viral genome and all HA, NA, and matrix (M) genes for virus subtype and influenza A virus identification have been published elsewhere (14,17–19). PCR products were processed with 1% agarose gel electrophoresis and were purified by QIAquick PCR purification kit (OIAGEN). Sequencing was performed by using the H5 and N1 specific primers, and sequence data were edited following methods previously described (14,17,18).

### Statistical Analysis

For each identified bird species, geographic location and season were recorded. Bird species were divided into 3 groups: 1) resident birds (nonmigratory populations), present year-round in Thailand; 2) migratory (visitor) birds, bird populations moving between Russia or China to Thailand during September/October and March/April; and 3) breeding visitor birds, which migrate to Thailand for breeding in different periods of the year.

To study the relevance between the regions and subtype H5N1 outbreaks in wild birds, we divided Thailand into 4 major geographic regions (northern, northeastern, central, and southern) on the basis of the former administrative region grouping system used by the Ministry of Interior, Thailand. Because of the high number of outbreaks in the Central region (14,17,20), it was further divided into 6 parts: central–northwest, central–north, central–central, central–east, central–southeast, and central–southwest. On the basis of procedures established by the Thai Meteorological Department, the seasons were divided into 3 periods: summer (March–June), the rainy season (July–October), and winter (November–February).

Prevalence of HPAI (H5N1) infection and 95% confidence intervals (CIs) were calculated for each group of bird species, sampling region, and season. Three variables associated with HPAI (H5N1) prevalence were analyzed by binary logistic regression. Overall significance of the model was assessed by the likelihood ratio χ^2^ test. The goodness-of-fit was calculated by using the Hosmer-Lemeshow goodness-of-fit test. Statistical significance of the regression coefficients was tested by using the Wald likelihood ratio test. Odds ratios (OR) and respective 95% CI were calculated. For multiple comparisons, the Bonferroni multiple comparison correction was applied to demonstrate statistical significance (p<0.001). Statistical analysis was performed by using statistical software SPSS version 17 (SPSS Inc., Chicago, IL, USA).

Data on outbreaks among poultry were taken from Tiensin et al. (16). We used their definition of poultry, which encompasses all farmed avian species in Thailand, including backyard chickens and ducks. Different species or types of production systems were not differentiated in the data. Using a nonhomogeneous birth model (21), we investigated the association between subtype H5N1 presence in infected poultry flocks and wild birds. Prevalence data from the 9 different regions were modeled independently and conditioned on the number of infected birds during the first month of detected infection for each region. Time lapse was measured in months from the first month infection was detected. To analyze the association between presence of subtype H5N1 in wild birds and outbreaks in poultry, we pooled data for the 3 wild bird groups (resident birds, migratory visitor birds, and breeding visitor birds) to increase power.

In most regions, sampling among wild birds was only done systematically after a poultry outbreak in that region, except in the central–northwest, central–north, and central–central regions. We could therefore only use the latter 3 regions to investigate whether the presence of infected wild birds was related to the poultry outbreak.

The nonhomogeneous birth model depends on the so-called reproductive power, which statistically quantified (in our setting) the ability of infected poultry flocks to spread infection to susceptible poultry flocks. For the statistical model, we used probability distributions from the Burr family. Distribution functions Burr XII and Burr III were fitted by using a conditional fitting procedure (21). For every region, we determined whether infected wild birds were detected during a particular month. A wild-bird infected month was defined as a month in which there was detection of infected wild birds or which showed wild-bird infection in the preceding month. We investigated whether wild-bird infection affected the reproductive power for the poultry outbreak in the same region. Reproductive power for wild-bird infected months was compared with that in non–wild-bird infected months for the central–northwest, central–north, and central–central regions. For comparison, we also calculated the reproductive power for poultry outbreaks for the 6 other regions of Thailand by using previously described methods (22). Model selection was done by using Akaike’s Information Criterion (AIC) (www.modelselection.org/aic).

## Results

### Descriptive Statistics

**Figure A1:**
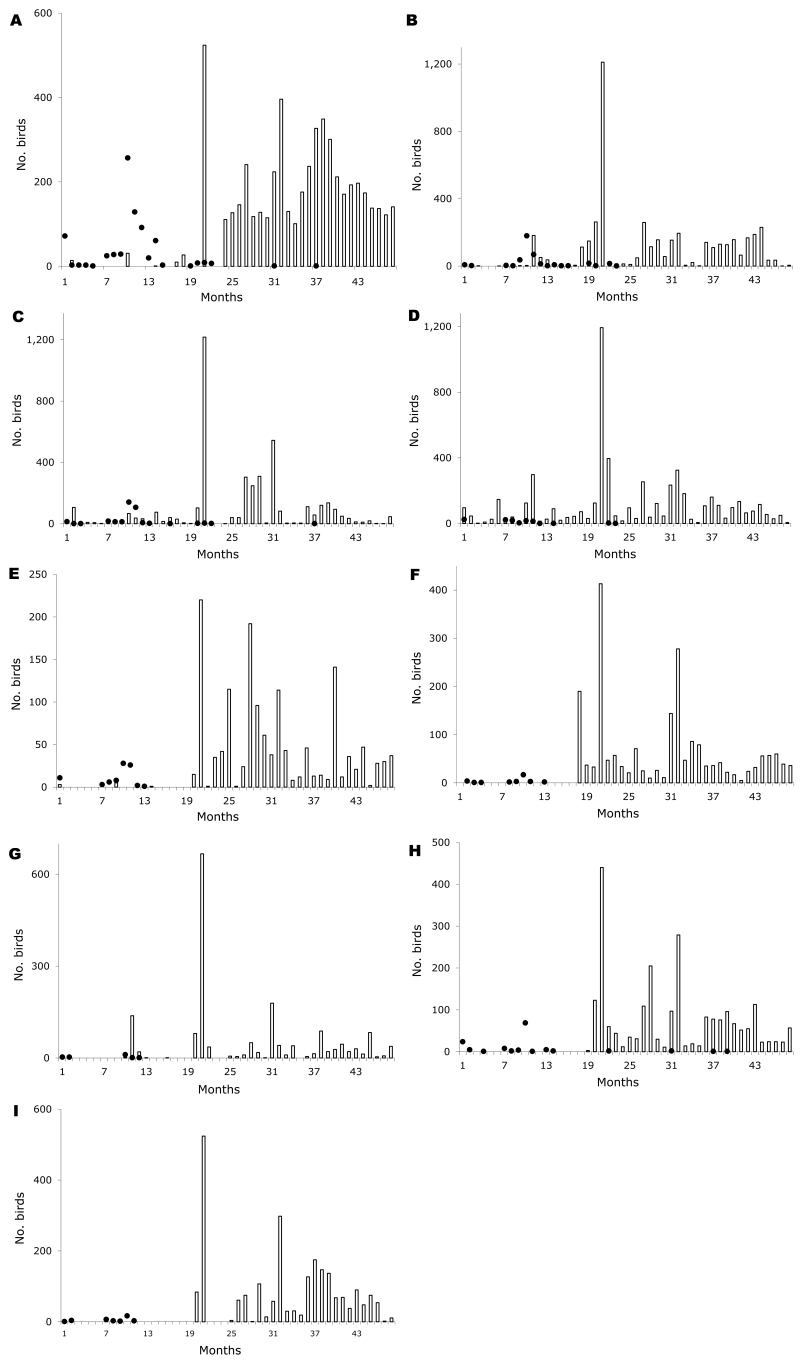
The number of sampled wild birds per month (bars) and the number of highly pathogenic avian influenza (H5N1) virus-infected poultry flocks (dots) in 9 regions of Thailand, 2004–2007.

Infected poultry flocks and wild birds were found in all 9 regions during the study period. In Figure A1, we present the numbers of wild birds sampled per month for each of the 9 regions and outbreak data of subtype H5N1 in poultry flocks. A total of 24,712 wild birds were sampled, consisting of 303 species, 64 families, and 20 orders (Table A1). Of these, 192 samples were positive for subtype H5N1, resulting in an overall prevalence of 0.78% (95% CI 0.67%–0.89%) (Table A1). Positive samples were found in 35 species of 12 orders (Table A2). Prevalence differed significantly among the group of wild bird species (p<0.001), with a prevalence of 0.187% (95% CI 0.01%–0.21%) in migratory birds (n = 2,142), 0.829% (95% CI 0.66%–0.94%) in resident birds (n = 16,633), and 0.814% (95% CI 0.61%–0.99%) in breeding visitor birds (n = 6,143). The highest prevalence of virus-positive birds was found in resident and breeding visitor birds (p<0.001) (Table A3).

**Figure 1:**
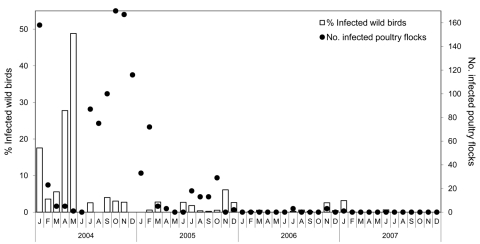
Epidemic curve of the number of highly pathogenic avian influenza (H5N1) virus infections in poultry flocks and percentage of infected wild birds during January 2004–December 2007, Thailand.

The aggregated data from Figure A1, presented for Thailand as a whole in Figure 1, show a marked increase in the number of infected poultry flocks detected from September through December 2004. A relatively high number of wild birds positive for subtype H5N1 were detected from January 2004 through May 2004, before the poultry outbreaks in June 2004. Infections in wild birds were consistently detected after the poultry outbreaks had ended, except during April and May in 2005, 2006, and 2007.

**Figure 2:**
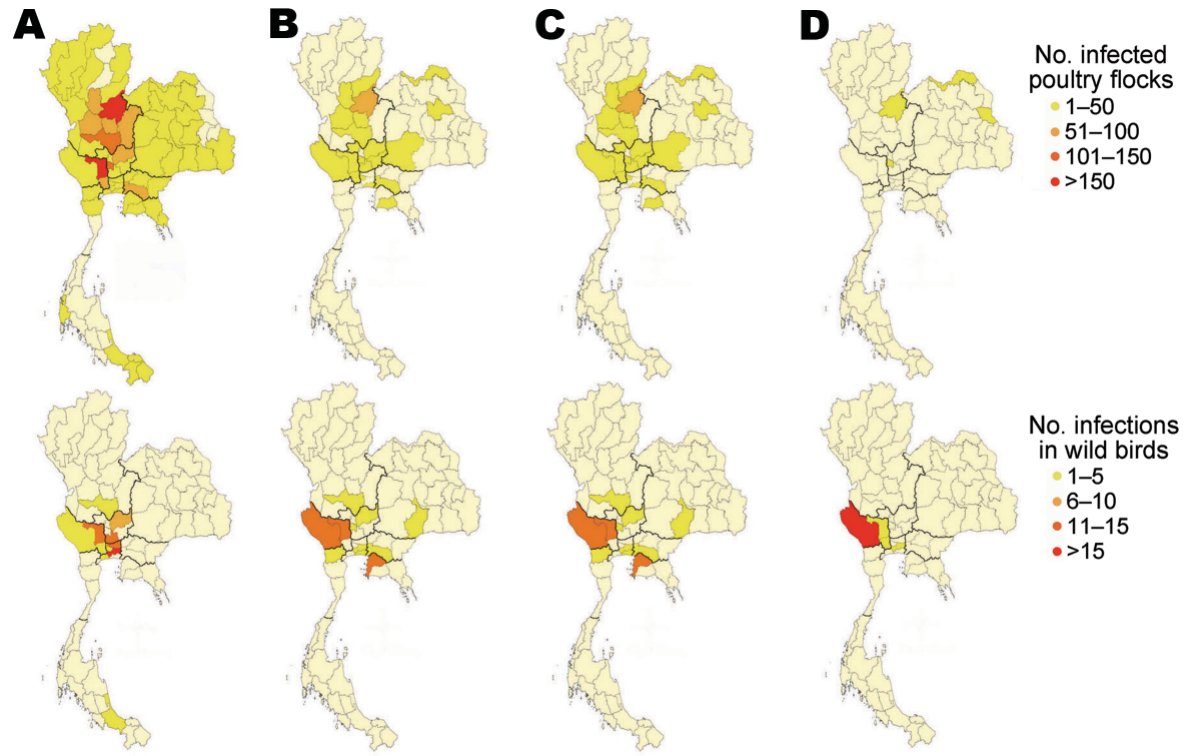
Distribution of highly pathogenic avian influenza (HPAI) subtype H5N1 infections in poultry flocks (top) and wild birds (bottom), Thailand. A) 2004, B) 2005, C) 2006, and D) 2007.

The spatial distribution and size classes of infected poultry flocks, as well as numbers of infected wild birds detected, are shown in Figure 2. In 2004 and 2005, infected wild birds were reported in the same locations where infected poultry flocks were found, especially in the central region. No infected poultry flocks were found in 2006 and 2007 in these areas. Subtype H5N1 prevalence in wild birds differed by sampling location. Central Thailand had the highest overall prevalence of 0.9% (95% CI 0.77%–1.03%), compared with other regions (p<0.001); the Northwest-Central region in central Thailand had a significantly higher prevalence (p<0.001) (Table A3).

The percentages of wild birds positive for subtype H5N1 in each season are also shown in Table A3. Prevalence differed significantly during January and February 2004 (7.92%; 95% CI 5.8–10.4; p<0.001) and in the summer of 2004 (11.79%; 95% CI 8.7%–15.8%; p<0.001), compared with the other seasons.

### Association between Outbreaks in Poultry and Infection in Wild Birds

**Figure A2:**
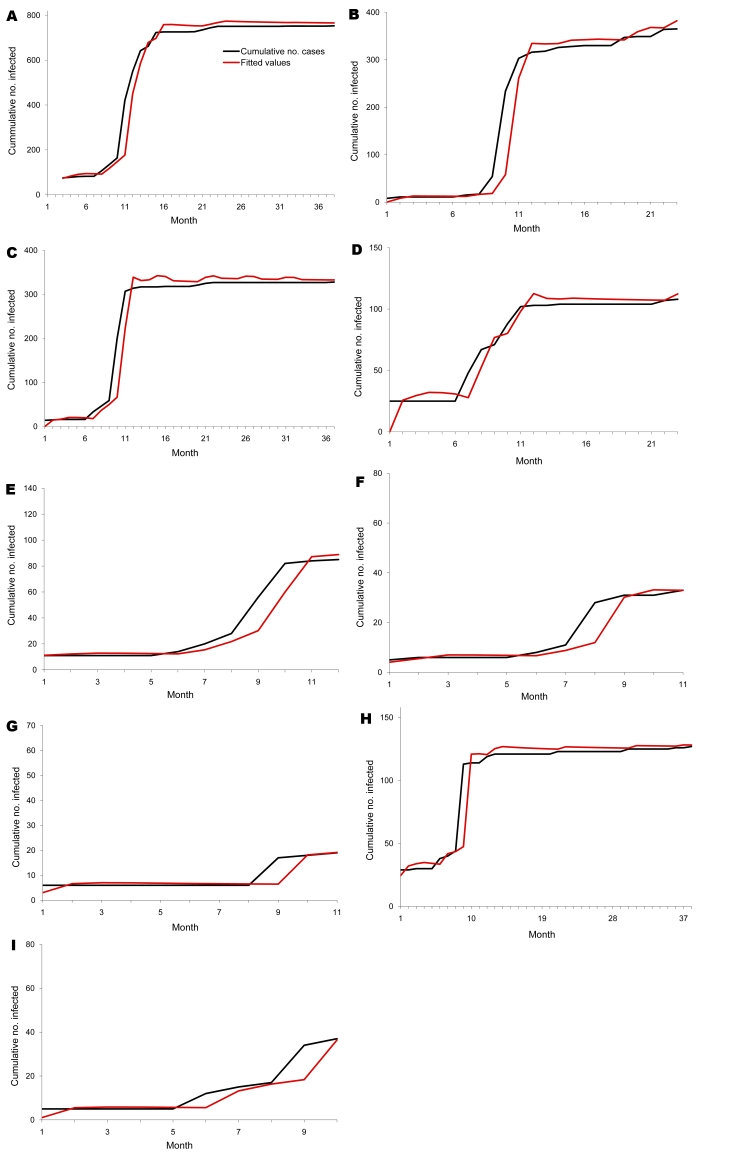
Outbreak data for highly pathogenic avian influenza (H5N1) infection in poultry, 2004–2007, Thailand. The cumulative number of cases and the fitted values were obtained by using the Burr XII model in the 9 regions.

The Burr XII and Burr III distributions each have 5 parameters. These distributions were used to model the observed poultry outbreak data for each of the 9 regions, taking into account wild-bird infection. The AIC, when we used the Burr XII model to fit the observed data, was 5,628.6, substantially lower than that for the Burr III distribution, which gave an AIC of 5,829.8. We therefore chose the Burr XII distribution to model the data (Figure A2 gives the fit to the data for all 9 regions). The model fits the data rather well.

**Figure 3:**
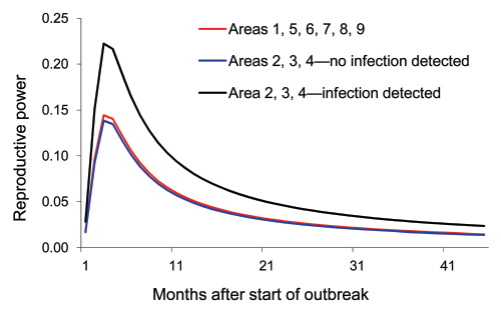
Reproductive powers of highly pathogenic avian influenza (H5N1)–infected poultry flocks in wild-bird infected months and in non–wild-bird infected months within different regions of Thailand, 2004–2007.

We also fitted the Burr XII distribution to model the observed poultry outbreak data in non–wild-bird infected months, leading to an AIC of 5,677.7. Because the model with wild-bird infection has a lower AIC, data clearly show that the reproductive power of poultry flocks in wild-bird infected months was higher than in non–wild-bird infected months. Parameter estimates for the model are shown in the Table. The log of the proportionality (ln[*c_3_*]) is 0.523, corresponding to a proportionality factor of ≈1.67, indicating that the reproductive power in wild-bird infected months is ≈1.7× higher than that in non–wild-bird infected months (Figure 3, where we give the reproductive power for the associated period). In Figure 3, we have also plotted the reproductive power for the 6 regions for which we could not do the wild-bird related comparison (regions 1 and 5–9). The reproductive power as a function of time was almost indistinguishable from the curve for the non–wild-bird infected months in regions 2, 3, and 4.

**Table:** Parameter estimation of the nonhomogeneous birth model using the Burr XII distribution for documenting data on HPAI (H5N1) outbreak in poultry, Thailand, 2004–2007*

Parameter	Estimate	SE
In(*b_1_*)	0.772	0.0777
In(*a*)	1.142	0.0627
In(*c_1_*)	−1.574	0.0746
In(*c_2_*)	−0.045	0.0627
In(*c_3_*)	0.523	0.073

*HPAI, highly pathogenic avian influenza.

## Discussion

We analyzed one of the largest datasets available of wild birds sampled for HPAI (H5N1) infection in Thailand, a country where several outbreaks of the disease have occurred in poultry flocks. Our aim was to determine the prevalence and distribution of HPAI (H5N1) in wild birds and to determine whether an association exists between outbreaks in poultry flocks and in wild birds within different regions in Thailand. We calculated the reproductive power in poultry flocks, a measure for the ability of a poultry flock to infect other susceptible poultry flocks. Notably, reproductive power was 1.7× higher in so-called wild-bird infected months, compared with poultry outbreaks in non–wild-bird infected months, suggesting a strong association of spread among poultry flocks and the presence of the infection in wild birds.

Poultry flocks in this study represent several avian species, which were considered as a single group with equal infectiousness, susceptibility, and other characteristics, in the absence of more precise information. Domestic ducks, which normally manifest a subtype H5N1 infection subclinically, were included in the poultry group. Ducks were not sampled according to criteria related to clinical signs. Available data do not allow a more differentiated analysis.

To quantify the association with outbreaks in poultry, we regarded wild birds as 1 group. We can therefore not differentiate the quantification of interaction to the level of specific wild-bird groups. In our additional analyses, however, most cases of HPAI (H5N1) infection in wild birds were found in resident birds, as compared with migratory and breeding visitor birds. Therefore, resident wild birds may be responsible for the association that we quantified. Our results can possibly be explained by the difference in exposure time of the wild birds. We partially confirmed, but more importantly expanded and added detail to, the conclusions reached by Siengsanan et al. (14), on the basis of pooled samples for a smaller part of the database. Bird species seemed to differ in susceptibility for infection. In our study, H5N1 virus infection was detected in many resident bird species, but we did not have a sufficient number of birds to differentiate in the quantitative analysis between different species. Species do differ, however, in terms of potential contact to poultry, especially birds considered to be peridomestic species of the Columbiformes, Cuculiformes, and Passeriformes orders, which are commonly associated with poultry environments. Transmission of subtype H5N1 to poultry populations by this group of resident bird species is more likely than transmission by other resident birds, including those belonging to the Galliformes, Gruiformes, Piciformes, Psittaciformes, and Struthioniformes orders. The habitats of these birds are not located near poultry areas. Previous experimental studies have shown that infected individuals of peridomestic species such as sparrow and starling can shed subtype H5N1 after infection, but they die quickly (23,24). Therefore, these birds are unlikely to be long-term reservoirs, but may be a higher risk to poultry than other resident bird species. Pigeons were found to be less susceptible to severe neurologic signs and death from HPAI (H5N1) infection (24). Infected pigeons appeared to shed low amounts of virus, thereby limiting virus transmission to sentinel birds (23–29). Our data showed a relatively high prevalence of HPAI (H5N1) in herons and storks (commonly known as scavengers and hunters of juvenile aquatic birds), which suggests that these birds are predominantly infected by contact with infected poultry flocks.

The prevalence of HPAI (H5N1) infections in resident birds was higher in areas with poultry flocks. We could not determine whether wild birds became infected because of spillover from poultry flocks or whether wild birds were the origin of outbreaks in poultry flocks. The association we found is not necessarily one of cause and effect. The 2 populations may have been affected by the same factors that increase transmission between flocks, e.g., contaminated water, movement between poultry flocks, or even increased transmission through fomites.

Even though data results are from the largest sampling effort available, the lack of a clear sampling strategy in the collection of wild-bird data precludes a definite answer to whether poultry flocks were infected with HPAI (H5N1) from infected wild birds or vice versa. Siengsanan et al. (14) suggested that poultry outbreaks precede detection of the infection in wild birds, but we have found no evidence either for or against that claim, again because of the sampling strategy used. One could argue the fact that infected poultry flocks produce massive amounts of virus, which supports the view that infection in wild birds is mostly seeded from poultry. A study carried out by Bavinck et al. (29) suggested that small backyard flocks did not contribute to the spread of subtype H7N7 infection in the Netherlands during 2003.

Seasonal bird migration, as well as enhanced movement and trade of poultry in the winter period caused by major social events occurring at the end of the year, may play a role in virus spread (30). Our data show increased prevalence among wild birds in all winter periods, with the exception of 2007 in which neither poultry farm outbreaks nor wild bird infections were detected. The actual sources of new introductions of HPAI (H5N1) into the commercial poultry flocks in Thailand could not be elucidated by our analysis.

From January through October 2004, a relatively small number of wild-bird samples was collected, compared with the number of samples collected from November 2004 to December 2007. Selection bias may have occurred during this period. Despite a bias in sampling numbers, HPAI (H5N1)–infected wild birds were detected during April–May 2004 just before the onset of the 2004 outbreak, but were not observed in that same period during 2005–2007 despite larger sampling numbers.

Variation in geographic distribution of HPAI (H5N1) infections in wild birds was observed over different areas. The central region of Thailand with dense poultry populations and large populations of birds living in the surrounding wetlands can be considered a hotspot for HPAI (H5N1) outbreaks. Our dataset shows high prevalence rates of the virus in the central region, corresponding with previous studies of HPAI (H5N1) surveillance in wild birds (14), in poultry flocks during 2004–2005 (16,17), and in cases of HPAI (H5N1) infection among humans during 2004 (31).

Associating these observations to our statistical model is interesting, because the reproductive power of poultry flocks in regions 1, 5, 6, 7, 8, and 9 was almost identical to that in regions 2, 3, and 4 during non–wild-bird infected months (Figure 3); regions 1, 5, 6, 7, 8, and 9 experienced no outbreaks in wild birds. It is however impossible to conclude from the current data that absolutely no wild birds were infected because, in these regions, relatively few samples were collected during the appropriate periods (Figure A1).

By determining the reproductive power in poultry, which is the ability of infected poultry flocks to spread infection to susceptible poultry flocks, we quantified the association between wild bird infection and outbreaks in poultry. We also attempted to take the reproductive power in wild birds, during poultry-infected months, as our starting point. However, too few infected wild birds were available for a reliable analysis.

## Appendix

**Table A1:** Overview of wild bird samples collected during 2004–2007, Thailand*

Order	Family	No. species	No. samples	No. avian influenza (H5N1)–positive birds	Prevalence (95% CI)
Anseriformes	*Anatidae*	8	300	2	0.007 (0–0.03)
Apodiformes	*Apodidae*	4	92	0	–
	*Hemiprocnidae*	1	6	0	–
Bucerotiformes	*Bucerotidae*	8	71	0	–
Caprimulgiformes	*Caprimulgidae*	1	3	0	–
Charadriiformes	*Charadriidae*	10	175	3	0.02 (0.01–0.04)
	*Jacanidae*	2	63	1	–
	*Laridae*	1	117	1	–
	*Recurvirostridae*	1	208	0	–
	*Rostratulidae*	1	6	0	–
	*Scolopacidae*	15	907	0	–
	*Sternidae*	2	12	0	–
	*Turnicidae*	2	21	0	–
	*Glareolidae*	1	10	0	–
Ciconiiformes	*Ardeidae*	23	2,181	10	0.15 (0.11–0.20)
	*Ciconiidae*	4	3,310	33	–
	*Thereskiornithidae*	2	19	0	–
Columbiformes	*Columbidae*	15	7,692	47	0.16 (0.12–0.21)
Coraciiformes	*Alcedinidae*	1	14	0	–
	*Coraciidae*	1	3	0	–
	*Halcyonidae*	5	112	0	–
	*Meropidae*	3	38	0	–
	*Upupidae*	1	4	0	–
Cuculiformes	*Cuculidae*	4	28	1	0.003 (0–0.01)
Falconiformes	*Accipitridae*	13	70	1	0.003 (0–0.01)
Galliformes	*Phasianidae*	25	695	28	0.09 (0.06–0.13)
Gruiformes	*Gruidae*	2	41	1	0.007 (0–0.03)
	*Rallidae*	4	93	1	–
Passeriformes	*Alaudidae*	1	6	0	0.19 (0.15–0.25)
	*Artamidae*	1	49	0	–
	*Campephagidae*	1	1	0	–
	*Cisticolidae*	3	236	0	–
	*Corvidae*	6	54	10	–
	*Dicruridae*	3	25	3	–
	*Estrildidae*	3	363	1	–
	*Fringillidae*	1	17	0	–
	*Hirundinidae*	6	952	0	–
	*Irenidae*	2	4	0	–
	*Laniidae*	2	4	0	–
	*Motacillidae*	7	213	0	–
	*Muscicapidae*	8	162	0	–
	*Oriolidae*	2	6	0	–
	*Passeridae*	3	2,997	17	–
	*Phylloscopidae*	1	1	0	–
	*Pittidae*	1	2	0	–
	*Ploceidae*	3	97	0	–
	*Pycnonotidae*	9	519	3	–
	*Rhipiduridae*	2	19	0	–
	*Sturnidae*	11	1,969	23	–
	*Sylviidae*	7	97	0	–
	*Timaliidae*	8	25	0	–
	*Zosteropidae*	2	2	0	–
Pelecaniformes	*Pelecanidae*	1	6	0	–
	*Phalacrocoracidae*	2	128	0	–
Piciformes	*Megalaimidae*	5	16	1	0.003 (0–0.01)
	*Picidae*	2	3	0	–
Psittaciformes	*Cacatuidae*	7	54	1	0.014 (0–0.03)
	*Chloropseidae*	1	3	0	–
	*Psittacidae*	26	201	3	–
Strigiformes	*Strigidae*	2	14	0	–
	*Tytonidae*	1	1	0	–
Struthioniformes	*Struthionidae*	1	150	1	0.003 (0–0.01)
Podicipediformes	*Podicipedidae*	1	5	0	–
Casuariiformes	*Casuariidae*	1	20	0	–
Total: 20	64	303	24,712	192	(0.67–0.89)

*CI, confidence interval; –, not applicable.

**Table A2:** Highly pathogenic avian influenza virus (H5N1) infection in wild birds, 2004–2007, Thailand*

Order	Common name	Species	Type of bird	No. case(s)/total no. sampled			
				2004	2005	2006	2007
Anseriformes	Lesser whistling duck	*Dendrocygna javanica*	Breeding visitor	1/11	0/1	1/42	NS
Ciconiiformes	Asian openbill strok	*Anastomus oscitans*	Breeding visitor	20/61	7/1,513	6/1,154	NS
	Heron	*Egretta spp.*	Breeding visitor	1/92	NS	3/303	NS
	Pond heron	*Ardeola grayii*	Breeding visitor	NS	NS	NS	6/12
Columbiformes	Rock pigeon	*Columba livia*	Resident	17/480	8/1,921	9/1,693	10/2,089
	Zebra dove	*Geopelia striata*	Resident	1/27	NS	NS	NS
	Emerald dove	*Chalcophaps indica*	Resident	NS	1/1	NS	NS
	Dove	*Streptopelia spp.*	Resident	NS	NS	1/174	NS
Charadriiformes	Pheasant-tailed jacana	*Hydrophasianus chirurgus*	Breeding visitor	1/1	NS	NS	NS
	Brown headed gull	*Chroicocephalus brunnicephalus*	Migratory	NS	1/22	NS	NS
	Kentish plover	*Charadrius alexandrinus*	Migratory	NS	NS	3/12	NS
Cuculiformes	Common koel	*Eudynamys scolopaceus*	Resident	NS	NS	1/3	NS
Falconiformes	Black kite	*Milvus migrans*	Breeding visitor	NS	1/8	NS	NS
Galliformes	Green peafowl	*Pavo muticus*	Resident	1/8	4/19	NS	NS
	Jones silver pheasant	*Lophura nycthemera jonesi*	Resident	4/4	3/42	NS	NS
	Golden pheasant	*Chrysolophus pictus*	Resident	1/1	1/15	NS	NS
	Crested partridge	*Rollulus rouloul*	Resident	2/3	NS	NS	NS
	Ring necked pheasant	*Phasianus colchicus*	Resident	1/1	2/8	NS	NS
	Lewis silver pheasant	*Lophura nycthemera nycthemera*	Resident	NS	3/13	NS	NS
	Kilij pheasant	*Lophura leucomelanos*	Resident	NS	6/35	NS	NS
Gruiformes	White breasted waterhen	*Amaurornis phoenicurus*	Resident	1/16	NS	NS	NS
	Sarus crane	*Grus antigone*	Resident	NS	1/12	NS	NS
Passeriformes	Streak eared bulbul	*Pycnonotus blanfordi*	Resident	3/11	NS	NS	NS
	Europian tree sparrow	*Passer montanus*	Resident	1/192	9/1,723	NS	6/654
	Large billed crow	*Corvus macrorhynchos*	Resident	10/12	NS	NS	NS
	Common myna	*Acridotheres tristis*	Resident	6/77	3/372	NS	6/85
	Black drongo	*Dicrurus macrocerus*	Breeding visitor	NS	NS	3/6	NS
	Asian pied starling	*Gracupica contra*	Resident	NS	NS	3/19	1/23
	White vented myna	*Acridotheres grandis*	Resident	NS	NS	NS	4/91
	Scaly breasted munia	*Lonchura phuctulata*	Resident	NS	NS	NS	1/23
	Plain backed sparrow	*Passer flaveolus*	Resident	NS	NS	NS	1/4
Piciformes	Great barbet	*Megalaima virens*	Resident	NS	1/2	NS	NS
Psittaciformes	Cockatoo	*Psittacus novaehollandiae*	Resident	1/5	NS	NS	NS
	Blossom headed parakeet	*Psittacula roseate*	Resident	NS	3/4	NS	NS
Struthioniformes	Ostrich	*Struthio camelus*	Resident	1/69	NS	NS	NS

*NS, not sampled.

**Table A3:** Prevalence of highly pathogenic avian influenza (H5N1) infection in wild birds, 2004–2007, Thailand *

Characteristic	No. infected birds	No. tested birds	Prevalence, % (95% CI)
Type of bird			
Migratory	4	2,142	0.187 (0.01–0.21)
Resident	138	16,633	0.829 (0.66–0.94)
Breeding visitor	50	6,143	0.814 (0.61–0.99)
Regions			
North	13	5,321	0.24 (0.1–0.3)
Central-Northwest	77	4,510	1.71 (1.38–2.02)
Central-North	30	4,075	0.74 (0.49–0.91)
Central-Central	54	5,327	1.01 (0.78–1.22)
Central-East	1	1,462	0.07 (0.02–0.32)
Central-Southeast	21	2,083	1.01 (0.64–1.36)
Central-Southwest	3	1,713	0.18 (0–0.23)
Northeast	5	2,298	0.22 (0.05–0.35)
South	8	2,348	0.34 (0.11–0.49)
Seasons, y			
Winter (January –February), 2004	29	366	7.92 (5.8–10.4)
Summer (March–June), 2004	27	229	11.79 (8.7–15.8)
Rainy (July–October), 2004	9	359	2.51 (1.51–4.32)
Winter (November–December), 2004 and (January–February), 2005	19	1,027	1.85 (1.29–2.70)
Summer (March–June), 2005	12	611	1.96 (1.26–3.15)
Rainy (July–October), 2005	25	8,026	0.31 (0.22–0.43)
Winter (November–December), 2005 and (January–February ), 2006	19	1,317	1.44 (1.00–2.10)
Summer (March–June), 2006	7	3,768	0.19 (0.11–0.32)
Rainy (July–October), 2006	17	4,528	0.38 (0.25–0.56)
Winter (November–December), 2006 and (January–February), 2007	44	3,261	1.35 (1.06–1.73)
Summer (March–June), 2007	4	3,032	0.13 (0.06–0.30)
Rainy (July–October), 2007	0	2,338	NA
Winter (November–December), 2007	0	652	NA

*CI, confidence interval; NA, not applicable.
