# Errata—Vol. 17, No. 6

**DOI:** 10.3201/eid1709.C31709

**Published:** 2011-09

The abstract of the article Wild Birds and Increased Transmission of Highly Pathogenic Avian Influenza (H5N1) among Poultry, Thailand (Juthatip Keawcharoen et al.) incorrectly referred to the swab samples collected. The article has been corrected online (www.cdc.gov/eid/content/17/6/1016.htm).

